# Role and mechanism of AT1-AA in the pathogenesis of HELLP syndrome

**DOI:** 10.1038/s41598-017-18553-x

**Published:** 2018-01-10

**Authors:** Shurui Bu, Yuxian Wang, Shuqing Sun, Yanqian Zheng, Zhu Jin, Jianming Zhi

**Affiliations:** 1 Department of Infectious Diseases, Jinshan Hospital, Fudan University, Shanghai, 201508 China; 2 Department of Obstetrics, The First Hospital, Shanxi Medical University, Taiyuan, Shanxi 030001 China; 3 Department of Critical Care Medicine, Weifang People’s Hospital, Weifang, Shandong 261041 China; 4 Department of Physiology, School of Medicine, Shanghai Jiao Tong University, Shanghai, 200025 China

## Abstract

HELLP syndrome remains a leading cause of maternal and neonatal mortality and morbidity worldwide, which symptoms include hemolysis, elevated liver enzymes and low platelet count. The objective of this study was to determine whether HELLP is associated with AT1-AA. The positive rate and titer of AT1-AA in plasma from pregnant women were determined, and the correlation of AT1-AA titer with the grade of HELLP was analyzed. A HELLP rat model established by intravenous injection of AT1-AA. Our experimental results show the AT1-AA titer and positive rate were significantly higher in HELLP group, and AT1-AA titer were positively correlated with the level of TNF-α and ET-1 in plasma and the grade of HELLP syndrome. The results of animal experiments showed that the typical features of HELLP in the pregnant rats after AT1-AA injection. The levels of TNF-α and ET-1 in plasma and liver tissue were significantly increased in AT1-AA-treated rats compared with control rats. The HELLP syndrome induced by AT1-AA was attenuated markedly after administration of losartan. These data support the hypothesis that one the potential pathway that AT1-AA induce damage to capillary endothelial cells and liver during pregnancy is through activation of TNF-α and ET-1.

## Introduction

HELLP (hemolysis, elevated liver enzyme levels and low blood platelet count) syndrome is an obstetric complication first reported by Weinstein in 1982. The incidence of HELLP syndrome accounts for 0.5–1% of all pregnancies. HELLP pregnant women can be complicated with pulmonary edema, placental abruption, coelomic fluid, postpartum hemorrhage, disseminated intravascular coagulation (DIC), renal failure, and liver rupture, causing a high mortality rate between 7.4% and 34%^1^. In addition, HELLP may impose great impact on the fetus, including growth retardation, fetal death, stillbirth and premature birth due to insufficient placental blood and oxygen supplies, and decreased placental function. According to the summary on the course of pregnancy made by Murray *et al*.^2^, 85% of their 20 pregnant women underwent Cesarean section within 24 hours after the confirmed diagnosis of HELLP, including 65% premature births with a mean gestational age of 33.5 weeks and a mean birth weight of 1923 g, of which 40% fetuses suffered from respiratory distress syndrome (RDS).

The main pathological changes of HELLP are similar to those of gestational hypertension, including vascular spasms, vascular endothelial injury, platelet aggregation and consumption, fibrin deposition, and end-organ ischemia. Although the initiation mechanism of HELLP remains unclear, many studies have provided more evidence to support the involvement of immune factors in HELLP. It is generally believed that maternal immune rejection against the fetus due to damage to the mechanism of maternal immune intolerance is the principal cause of HELLP. Numerous studies discovered that activation of complexes and elevation of anaphylatoxin C_3a_ and C_5a_, and terminal C_5b-9_ complement complex in the blood of HELLP patients could stimulate macrophages, white blood cells and platelets to synthesize vascular active substances, which induce spastic constriction of blood vessels, platelet aggregation and consumption due to endothelial injury, resulting in thrombocytopenia, hemolysis and elevation of liver enzymes^3–5^.

The renin-angiotensin system (RAS) is an important humoral regulation system in the human body. Angiotensin II (Ang II) is the regulatory peptide of this system that plays important roles in cell growth, development, proliferation and apoptosis, as well as in inflammatory response. In addition, abnormally increased angiotensin II plays a vital role in hypertension and kidney disease^6^. The pathophysiological roles of Ang II are associated with the activation of AT1 receptors. Research in recent years has discovered that agonistic autoantibody against type-1 angiotensin II receptor (AT1-AA) can exert an agonist-like effect through activation of AT1 receptors. This autoantibody can regulate the activity of intracellular PKC by acting on the AT1-receptor extracellular bicyclic peptide and may participate in Ang II-induced vascular lesions in patients with preeclampsia. However, the role of the AT1-receptor antibody in the pathogenesis of HELLP syndrome is uncertain.

The objective of the present study is to verify of AT1-AA participates in the pathogenesis of HELLP by detecting AT1-AA in the plasma of HELLP patients. In addition, we also made a correlation analysis on HELLP classification. Finally, we injected the AT1-AA solution extracted from the plasma of HELLP patients to pregnant rats of 10-day gestational age to see whether it could reproduce the HELLP syndrome for the sake of analyzing the potential mechanism.

## Results

### Clinical characteristics

The study was performed in 59 HELLP women (HELLP group) and 45 normotensive pregnant women (control group). The mean age of the two groups was 28.2 ± 5.6 (rang 23~41) and 27.4 ± 6.2 (rang 24~45) years, respectively. The blood pressure and level of urine protein in HELLP group were significantly higher than those in control group (Table 1). Platelet counts (PC) in HELLP group was significantly lower than that in control group (87.6–7.8 × 10^9^/L *vs*. 216–17.8 × 10^9^/L). According to PC, class I, II, III were observed in 24 (40.6%) cases, 19 (32.2%) cases, and 16 (27.2%) cases in HELLP group, respectively (Table 1). Meanwhile, alanine aminotransferase (ALT), aspartic aminotransferase (AST), lactate dehydrogenase (LDH), urea nitrogen (BUN), and creatinine (Cr) in HELLP group were significantly higher than those in control group (P < 0.05), but albumin (ALB) was significantly decreased (P < 0.05) in HELLP group. The biochemical indicators of the HELLP group and control groups are shown in Table 1.

**Table 1:** The various indicators of control and HELLP group (mean ± SD).

Parameter	Control (n = 45)	HELLP group			
		All patient (n = 59)	Class I (n = 24)	Class II (n = 19)	Class III (n = 16)
SBP (mmHg)	115 ± 10	154 ± 12**	157 ± 15	153 ± 15	150 ± 16
DBP (mmHg)	74 ± 8	109 ± 11**	112 ± 10	110 ± 10	103 ± 11
BPC (×10^9^/L)	216.4 ± 18.7	87.6 ± 8.2**	45.3 ± 4.2	81.5 ± 6.7^#^	125.6 ± 8.4^##^
ALB (g/L)	48.6 ± 6.3	25.8 ± 4.2**	22.6 ± 2.7	26.2 ± 4.5^#^	28.4 ± 3.8^##^
UP (mg/24 h)	NA	1.76 ± 0.52**	2.12 ± 0.61	1.72 ± 0.35^#^	1.25 ± 0.36^##^
ALT (IU/L)	41 ± 5	357 ± 63**	469 ± 57	346 ± 69^##^	202 ± 46^##^
AST (IU/L)	89 ± 24	921 ± 256**	1084 ± 264	983 ± 126^#^	603 ± 89^##^
LDH (IU/L)	459 ± 59	2052 ± 389**	2314 ± 329	2016 ± 425^#^	1701 ± 356^##^
BUN (mmol/L)	7.5 ± 3.2	14.7 ± 8.3**	16.8 ± 5.9	14.5 ± 6.6	11.8 ± 7.2^#^
BUA (μmol/L)	285 ± 56	502 ± 112*	513 ± 50	508 ± 58	478 ± 41
Cr (μmol/L)	92 ± 32	152.6 ± 57.7*	179.5 ± 45.3	147.6 ± 53.1^#^	117.2 ± 44.2^##^
ET-1 (pg/ml)	0.54 ± 0.12	1.02 ± 0.28**	1.28 ± 0.32	0.86 ± 0.16^##^	0.82 ± 0.24^##^
sFts-1 (pg/ml)	0.86 ± 0.34	1.65 ± 0.32**	2.04 ± 0.42	1.54 ± 0.36^##^	1.20 ± 0.32^##^

^*^P < 0.05. **P < 0.01 vs group C; ^#^P < 0.05. ^##^P < 0.01 vs Class I of HELLP, respectively.

### AT1-AA titer in HELLP syndrome

**Figure 1:**
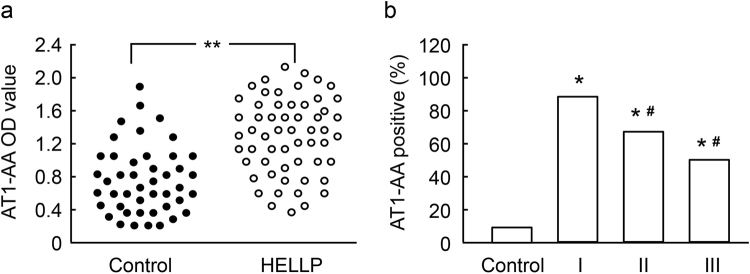
AT1-AA level in normal pregnant women and pregnant women with HELLP syndrome. AT1-AA titer was significantly increased in HELLP group (**a**). The number of AT1-AA positive patients was correlated with platelet count (**b**). Values are mean ± SD. *P < 0.01 *vs*. control group; ^#^P < 0.01 *vs*. class I of HELLP, respectively.

The positive rate and geometric mean titer (GMT) of AT1-AA were 64.4% and 1:172.6 ± 12.8 respectively in HELLP group, which is significantly higher than those in the control group (13.3% and 1:28.9 ± 6.4) (Fig. 1a). The correlation was analyzed between plasma AT1-AA and HELLP class. As Fig. 1b shows, the positive rate of AT1-AA increased with the severity of HELLP: 85.8% (20/24) in class I, 63.2% (12/19) in class II and 52.4% (6/16) in class 3. In addition, the systolic blood pressure (SBP) and diastolic blood pressure (DBP) were also significantly correlated with AT1-AA titer in HELLP group: 45.8% (11/24) in low-BP group (SBP < 150 mmHg, DBP < 100 mmHg), and 68.6% (24/35) in high-BP group (SBP > 150 mmHg, DBP > 100 mmHg) (Table 2).

**Table 2:** Correlations between plasma AT1-AA titer and different variables in HELLP group.

Parameter	Control group (n = 45)	HELLP group (n = 59)				
		Class I (n = 24)	Class II (n = 19)	Class III (n = 16)	Total	P
**Blood pressure (mmHg)**						
SBP < 150, DBP < 100		8	9	7	24	<0.05
SBP > 150, DBP > 100		16	10	9	35	
**AT1-AA titer**						
<1:40	29	4	7	10	21	<0.01
>1:40	14	6	4	2	12	
>1:160	2	7	5	2	14	
>1:640		7	3	3	13	

### Relationship between levels of ET-1 and TNF-α and AT1-AA titer

**Figure 2:**
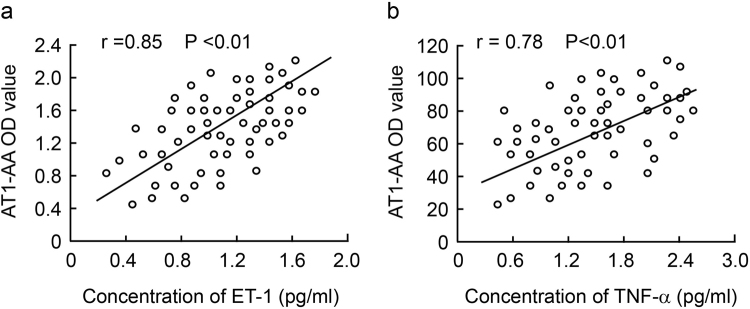
Scatter plots show a positive linear correlation between plasma ET-1 and TNF-α level and AT1-AA titer in pregnant women with HELLP syndrome. Values are mean ± SD.

As shown in Fig. 2, plasma levels of ET-1 and TNF-α in HELLP group were significantly higher more than those in the control group (1.02–0.28 pg/ml and 1.65–0.32 pg/ml *vs*. 0.54–0.12 pg/ml and 0.86–0.34 pg/ml, p < 0.001 and p < 0.01). There were significant differences in plasma ET-1 and TNF-α level between the different HELLP classes (P < 0.01). The findings of the study show that there was a significant positive correlation between the plasma level of ET-1 and TNF-α and the plasma AT1-AA titer in HELLP group (Fig. 2).

### Effect of AT1-AA on HELLP MAP and biochemistry

**Figure 3:**
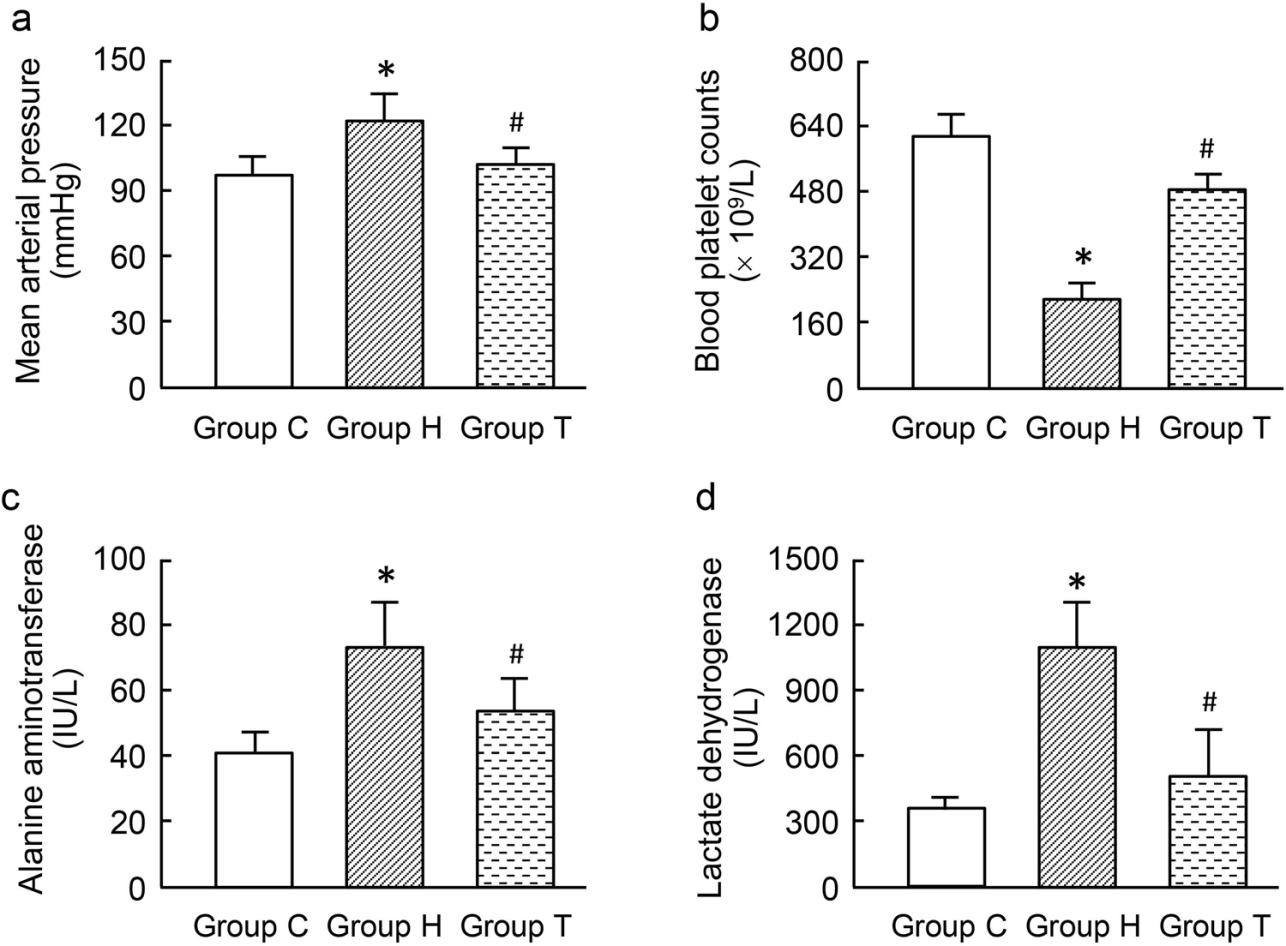
The mean arterial pressure, blood platelet count, alanine aminotransferase and lactate dehydrogenase in different rat groups. Values are mean ± SD. *P < 0.01 *vs*. group C; ^#^P < 0.01 *vs*. group H, respectively.

To analyze whether the AT1-AA could induce HELLP syndrome, AT1-AA was introduced into pregnant rats at gestation day (GD) 10 by tail vein injection. On the GD 11, the injection were repeated again (group H). Part of the pregnant rats infused with IgGs from the normal pregnant women served as controls (Group C). It was found that MAP in group H was significantly higher than that in group C (p < 0.01) (Fig. 3a). The total UP/Cr ratio in group H was significantly higher than that in group C (111 ± 16 mg/ml *vs*. 22 ± 1 mg/ml, P < 0.05) (Table 3). PC in group H was decreased significantly (p < 0.01) (Fig. 3b), but ALT and LDH were increased significantly as compared with those in group C (both p < 0.05) (Fig. 3c,d). Weight of the fetus and placenta in group H was lower than that in group C (P < 0.01), and the fetus height and tail length were significantly shorter than those in group C (P < 0.01). Compared with group C, the percentage of stillbirth (including absorbed fetuses) was significantly increased in group H (p < 0.05) (Table 3). There were no significant differences in liver (P = 0.42) or kidney (p = 0.71) weight between the two groups (Table 3). After treatment with losartan in group H (group T), there was no significant change in MAP (p = 0.45), UP (P = 0.11), PC (P = 0.21), ALT (p = 0.34) and LDH (p = 0.16) as compared with group C. However, PC in group T was decreased as compared with group C (p < 0.05).

**Table 3:** Maternal and fetal characteristics (mean ± SD).

Parameter	Group C	Group H	Group T
Pregnant rats initial weight (g, GD 12)	234 ± 6	231 ± 6	232 ± 5.9
Pregnant rats final weight (g, GD 20)	385 ± 12	359 ± 9*	376 ± 11^#^
Pregnant rats Initial MAP (mmHg, GD 12)	95.6 ± 4.6	96.1 ± 5.2	95.3 ± 4.2
Pregnant rats final MAP (mmHg, GD 20)	97.4 ± 5.6	124.1 ± 8.6**	101.4 ± 7.7^#^
Total urinary protein/creatinine	24.7 ± 3.2	108.2 ± 108.6**	32.8 ± 3.2^##^
Liver weight of pregnant rats (g, GD 20)	12.3 ± 0.45	13.1 ± 0.52	12.4 ± 0.48
Kidney weight of pregnant rats (g, GD 20)	0.72 ± 0.08	0.73 ± 0.08	0.74 ± 0.07
Number of live fetuses Medians (min.-max.)	12(10–15)	11(9–15)	12(10–15)
Percentage of Stillbirth (%)	1.4 (1/71)	10.3 (7/68)*	2.7 (2/74)^#^
Fetal weight (g, GD 20)	3.54 ± 0.25	3.08 ± 0.33**	3.38 ± 0.24^#^
Fetus height (cm, GD 20)	3.51 ± 0.14	3.24 ± 0.13**	3.48 ± 0.21^#^
Placenta weight (g, GD 20)	0.69 ± 0.08	0.58 ± 0.04*	0.66 ± 0.04^#^

^a^n = 6 for group C, group H and group T; ^b^n = 71 for group C; 65 for group H and 74 for group T. *P < 0.05. **P < 0.01 vs group C; ^#^P < 0.05. ^##^P < 0.01 vs group T, respectively.

### Change of ET-1 and TNF-α level in plasma and homogenate of the liver

**Figure 4:**
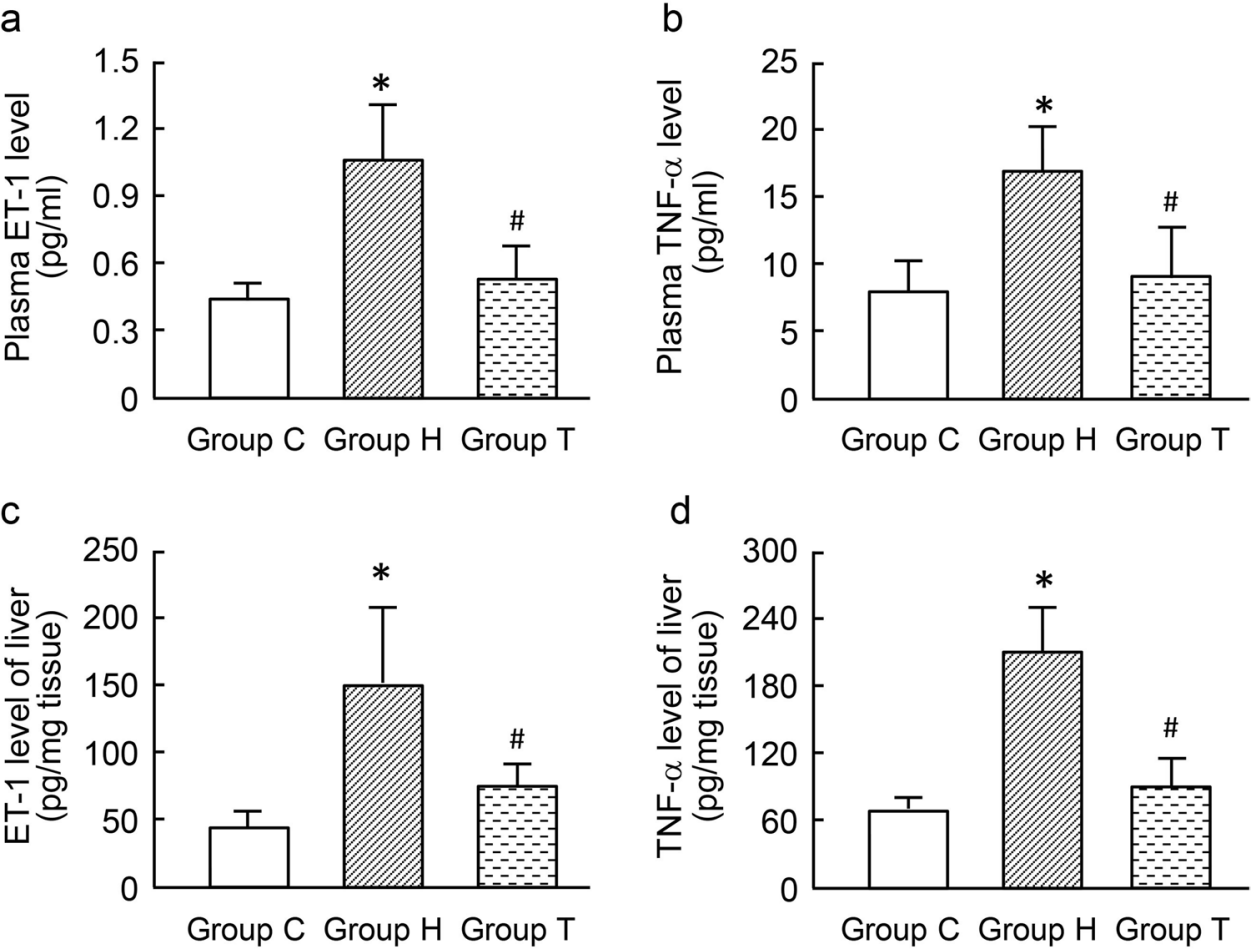
Levels of ET-1 and TNF-α in plasma and liver tissue of different groups of rats. *P < 0.01 *vs*. group C; ^#^P < 0.01 *vs*. group H, respectively.

On GD 20, ET-1 level in peripheral blood and homogenate of group H were significantly higher than those in group C. There was no significant difference in ET-1 between group T and H (Fig. 4). Change in TNF-α in peripheral blood and homogenate of group H was similar to ET-1.

### Histological observation and morphological change in experimental rats

**Figure 5:**
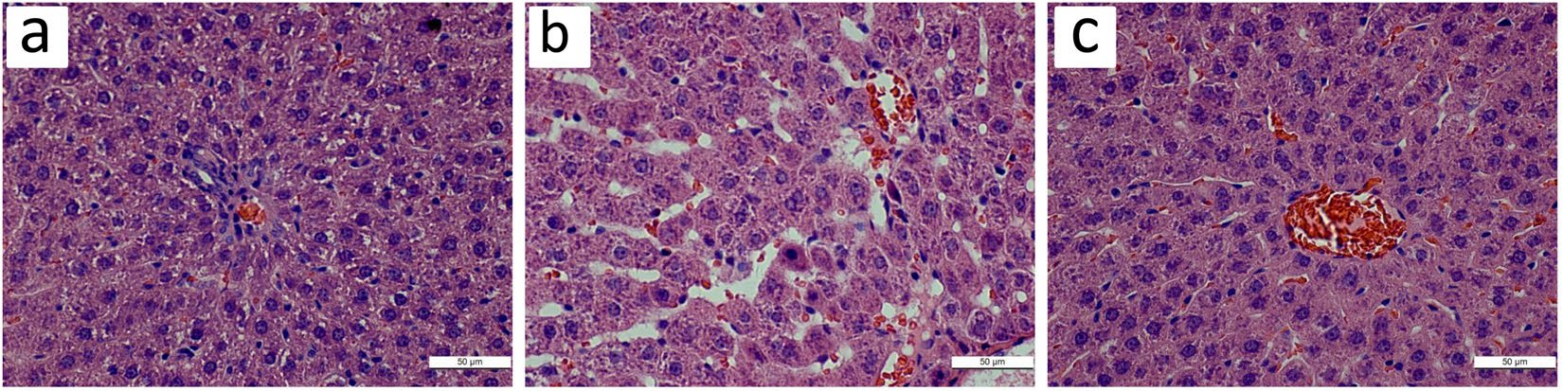
High resolution light microscopy. Representative histological images of the liver from group C (**a**), group H (**b**), group T (**c**). Original magnifications, 200.

HE staining showed that the structure of hepatic lobules and hepatocytes was normal in group C (Fig. 5a), and the structure of hepatic lobules in group H was basically normal except the presence of cell swelling, vacuoles in cytoplasm, and scattered, diffuse or localized hepatic steatosis with fat droplets predominating. The central venous sinus was obviously dilated and congested accompanied varying degrees of hepatocyte necrosis, and a small amount of lymphocyte infiltration was seen in the portal area (Fig. 5b). Compared with group H, the structure of hepatic lobules and hepatocytes was improved significantly in group T (Fig. 5c).

### Transmission electron microscopy (TEM)

**Figure 6:**
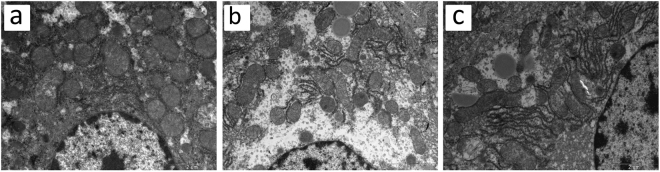
Transmission electron microscopy. Representative histological images of hepatic cells from group C (**a**), group H (**b**), group T (**c**). Original magnifications, 13500.

TEM showed regular morphology of hepatocytes in group C, with clear boundaries and abundant mitochondria and rough surfaced endoplasmic reticula; the mitochondrial cristae were normal with clear structures (Fig. 6a). In group H, the structure of hepatic cells was incomplete, with dilated and swollen mitochondria and broken mitochondrial cristae; the number of rough surfaced endoplasmic reticula was decreased; cells began undergoing fat degeneration, the plasma electron density was decreased, the content arrangement was sparse, and the plasma began undergoing large-area edematous morphological change; the number of lysosomes in hepatocytes was increased significantly (Fig. 6b). The ultrastructure of hepatocytes in group T was basically normal with a small number of swollen mitochondria (Fig. 6c).

**Figure 7:**
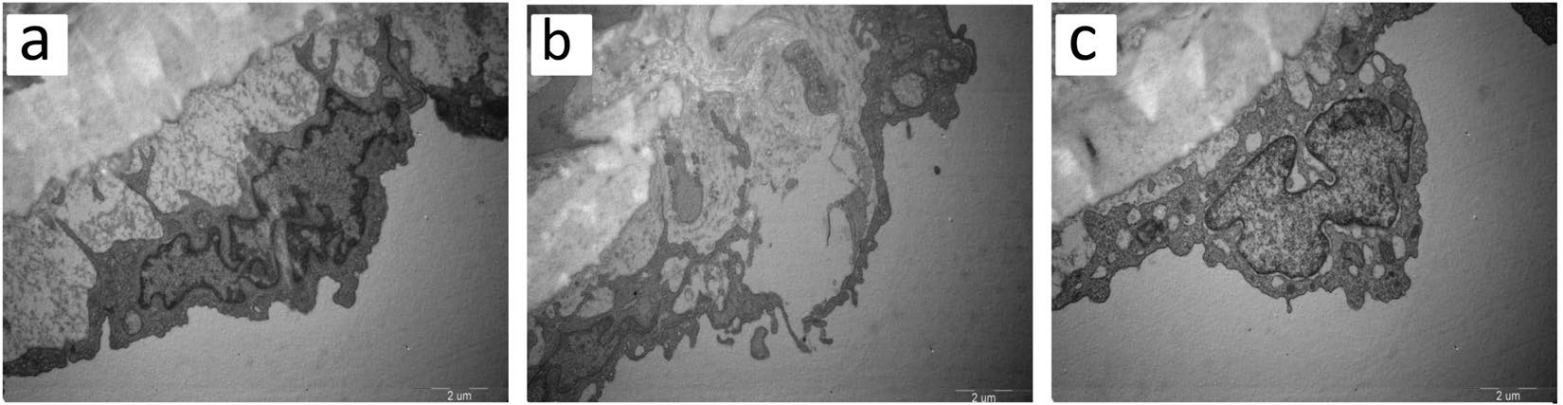
Transmission electron microscopy. Representative histological images of the aortic endothelial cells from group C (**a**), group H (**b**), group T (**c**). Original magnifications, 13500.

Thoracic aortic projection electron microscopy showed that the density of vascular endothelial cell membranes in group C was uniform, intact and continuous with clear structures and smooth surfaces; the plasma was abundant, and the morphology of mitochondria and rough surfaced endoplasmic reticula was normal; cells were connected closely with each other (Fig. 7a). In group H, the vascular endothelium was swollen and the surface was unsmooth; the number of vacuoles in the plasma was increased; most organelles were lysed and disappeared; mitochondria presented spherical shapes of different sizes, and the mitochondrial cristae were either deranged or even disappeared; the connection between cells was destroyed, and part of endothelium cells fell off (Fig. 7b). In group T, the endothelium was slightly edematous, the organelles were basically normal, and the connection between some of the endothelial cells was loosened (slacked) (Fig. 7c).

### The effect of AT1-AA on rat mitochondrial membrane potential

Rhodamine 123 is a cationic fluorescent dye that can penetrate the cell membrane and therefore is often used as an indicator to assess the intensity of mitochondrial membrane potential. In normal cells, Rhodamine 123 presents strong fluorescence when it binds with mitochondria charged with high negative membrane potential; when apoptosis occurs, the fluorescence of Rhodamine 123 becomes weak because its binding with mitochondria is reduced due to decreased depolarizing membrane potential. Our results indicate that Rhodamine retention in AT1-AA-treated hepatocytes decreased by 13.8% in 60 min, 24.6% in 90 min, and 37.4% in 120 min.

## Discussion

Preeclampsia and HELLP syndrome are idiopathic diseases originating from the placenta during pregnancy, constituting two main causes of maternal death, fetal death and premature birth. The main clinical manifestations of preeclampsia are hypertension and proteinuria after 20 weeks of gestational age, while the main clinical manifestations of HELLP are hemolysis, elevated liver enzymes and low PC. The association between the two entities remains unclear. Some studies reported that HELLP was the severe form of Preeclampsia (or eclampsia), while others argued that some HELLP patients only presented hypertension without proteinura, edema and other characteristic features of preeclampsia. Schutte analyzed^7^ deaths due to hypertensive disorders (with SPB ≥160 mmHg in all the women involved) and found that 60% of them died from preeclampsia (or eclampsia) and 52% died from thrombocytopenia (PC < 100 × 10^9^/L)^7^. We believe that HELLP syndrome is a serious multisystem disease involving the cardiovascular system, central nervous system, lung, liver, and kidney. In the present series of 59 HELLP patients, SBP was higher than 140 mmHg in 57 (96.7%) patients, including 32 patient with SBP >160 mmHg. Of the 59 HELLP patients, 25 (42.4%) presented the symptoms of preeclampsia.

Since the first detection of AT1-AA in the plasma of preeclampsia patients by Wallukat *et al*. in 1999, many studies have been performed to explore the association between AT1-AA and preeclampsia and found that AT1-AA was detectable in the plasma of about 50% of preeclampsia patients. Zhou *et al*. found that injection of pregnant mice with AT1-AA induced hypertension, proteinura, glomerular endothelial hyperplasia and other preeclampsia-like symptoms, and these symptoms could be inhibited by administration of AT1R antagonists^8^. In the present study, we also successfully copied the preeclampsia symptoms by injecting AT1-AAextracted from the serum of preeclampsia patients to rats of 13-day gestational age via the tail vein^9^. HELLP usually occurs during 32–34 gestational age. Bornstein *et al*. reported HELLP symptom can occur at a 16 weeks of gestation^10^. Fischer *et al*. reported a 24-year-lod woman with a twin pregnancy occurrence of HELLP in 18-week gestational age, and detected high-titer AT1-AA in the plasma^11^. In the present study, we detected AT1-AA in the plasma of 59 HELLP patients and found that the positive rate of AT1-AA was 76.3% (45/59), and 94.7% (22/24) in the most severe form of Class I.

Saito *et al*. proved empirically that patients who develop class 1 HELLP syndrome have significantly higher composite major maternal morbidity^12^. The pathological changes in HELLP are currently believed to be manifested by severe vascular endothelial injury and intra-luminal fibrin deposition. As a result, the physical substances flowing in the lumen are damaged when they contact with the affected part, leading to the formation of microemboli. Studies have revealed that abnormal expression of AT1-AA is closely associated with endothelial injury. Newton *et al*. found that AT-AA could increase endothelial permeability by activating AT1-R on endothelial cells^13^. It was found in our previous study that vascular endothelial cells became incomplete and deranged, and connections between them became larger with inflammatory cell infiltration after immunization of rats with the second extracellular loop of the AT1-receptor under the action of the antibody, which is similar to the finding of the present study that the endothelial function became abnormal due to inflammatory injury to vascular endothelial cells^14^. Abnormality of vascular constriction is another important pathological change in HELLP. Injury to the vascular endothelium will activate thrombocytes to secrete vasoconstrictors, thus increasing the constriction of vessels. Migration and proliferation of vascular smooth muscle cells (VMSCs) and synthesis of large amounts of extracellular matrix are the main reasons for intimal thickening, neointima formation and luminal stenosis, thus playing an important role in regulating vascular constriction. It was found in our previous study that AT1-AA exerted an effect similar to that of Ang II in inducing VSMC proliferation, vascular reconstruction, and the vascular endothelial injury^13^. As a result, vascular constriction is intensified and vascular relaxing response is decreased apparently, leading to end-organ injury and promoting the development and progression of multiple cardiovascular diseases. Some studies found that action of AT1-AA on AT1-R of VSMCs could directly increase vascular constriction and peripheral vascular resistance^15^. In addition, this action could amplify the vasoconstrictor action of Ang II by increasing the intracellular free Ca^2+^ level.

ET-1, a strong vasoactive substance mainly secreted from vascular endothelial cells, can regulate vascular homeostasis. Increased plasma ET-1 level is also an important cause for the occurrence of HELLP. The result of our experiment showed that the plasma ET-1 level was significantly elevated in HELLP patients. A recent study by Karakus *et al*.^16^ showed that the plasma ET-1 level in HELLP patients was significantly higher than that in preeclampsia patients and normal pregnant women. Bussen *et al*.^17^ reported that ET-1 elevation in pregnancy-complicated HELLP may be related to extensive vascular endothelial cell injury or dysfunction, and therefore could be used as an index of endothelial injury. Administration of exogenous ET-I could induce vascular spasms and ischemia necrosis of the liver, resulting in alterations of HELLP-related serum indexes^18^. Many studies^19,20^ reported that AT1-AA induced endothelial injury and release of ET-1 were one of the mechanisms underlying the pathogenesis of preeclampsia. Our experiment demonstrated that plasma ET-1 level in HELLP group was significantly increased more than those in the those control group. The plasma concentration was increased markedly after injection of high-titer AT1-AA in pregnant rats, and this phenomenon could be inhibited by administration of AT1-R antagonists. We therefore postulate that AT1-AA-induced HELLP-like syndrome induced by AT1-AA may be associated with the elevation of ET-1.

Some study results^21,22^ showed that the serum level of TNF-α in women with HELLP syndrome and model rats was elevated. TNF-α is a multifunctional cytokine that plays an important role in inflammation and immunity, as well as in the control of cell proliferation, differentiation and apoptosis. Several studies have demonstrated that there is a close relationship between the AT1-AA and TNF-a. Irani *et al*.^23^ confirmed that AT1-AA, through AT1 receptor–mediated TNF-α induction, contributed to increased soluble fms-like tyrosine kinase 1, soluble endoglin secretion, and thereby contributed to the pathophysiology of preeclampsia. Chronic over-expression of TNF-α may promote cell apoptosis and many other pathological processes induced by AT1-AA^24^. Another manifestation of HELLP is the elevation of serum liver enzymes due to death of hepatocytes^25^. On the one hand, cytokines such as TNF-α cause death of hepatocytes directly, and on the other hand, high plasma concentrations of AT1-AA, ET-1, thromboxane and other vasoconstrictors promote vascular constriction, causing blood flow obstruction in the hepatic sinus, which indirectly induces swelling and necrosis of hepatocytes.

## Methods

### Ethics statement

All human sample acquisitions were approved by the ethical committee of Shanghai Jiao Tong University School of Medicine, China, and performed in accordance with the declaration of Helsinki Principles. All participants provided written informed consent which was obtained before enrolment in the study. All animal experiments were performed according to the protocol approved by Shanghai Jiao Tong University School of Medicine Animal Care and Use Committee and in direct accordance with Ministry of Science and Technology of the People’s Republic of China on Animal Care guidelines. The protocol was approved by Shanghai Jiao Tong University Animal Care and Use Committee. Animals were sacrificed after anesthetized and all efforts were made to minimize animal suffering.

### Patient selecting

HELLP patients (HELLP group, n = 59) was diagnosed when individuals had thrombocytopenia (<150 × 10^9^/L) and increased lactate dehydrogenase (LDH, >600 IU/L) and aspartate transaminase (AST, >70 IU/L). According to the Mississippi classification, the severity of HELLP syndrome was divided into three categories: Class I (severe thrombocytopenia): platelets under ≤50 × 10^9^/L; Class II (moderate thrombocytopenia): platelets between 50 × 10^9^/L ~ 100 × 10^9^/L; Class III (AST > 40 IU/L, mild thrombocytopenia): platelets between 100 × 10^9^/L ~ 150 × 10^9^/L. Control pregnant women were selected on the basis of having an uncomplicated, normotensive pregnancy with a normal term delivery (control group, n = 45). All individuals with no previous history of hypertension, diabetes mellitus, vasculitis, renal disease are reported previously.

### Detection of biochemical indicators by ELISA assay

Blood samples collected from the patients and controls by forelimb vein were placed in pre-cooled tubes containing EDTA. About 2 ml blood sample was used to perform full blood count using an automated hematology analyzer. Part of the blood sample was used to implement blood count and peripheral smear to exclude pseudo-thrombocytopenia, and detect icroangiopathic features. The remaining sample was centrifuged at 2000 rpm for 20 min to collect the supernatant for measurement of alanine aminotransferase (ALT), AST, LDH, blood urea nitrogen (BUN), blood uric acid (BUA), creatinine (Cr) and levels of serum albumin (ALB). Enzyme-linked immunosorbent assay (ELISA) was adopted to analyze plasma AT1-AA titer as described previously^26^. The positivity of the sera was defined as P/N ≥2.1 (PN = specimen O.D. – blank O.D./negative control O.D. - blank control O.D.). The antibody titre was determined by the continuous double dilution of the samples from 1:20 and expressed as the maximum dilution when P/N ≥2.1. Expressing the results as a P/N ratio but not as an absolute O.D. value is considered to be advantageous when it comes to reducing the possible systemic errors. The plasma levels of endothelin-1 (ET-1) and tumor necrosis factor-alpha (TNF-α) were determined using ELISA. Whole blood was collected in EDTA treated tubes and processed for LDH, platelet count (PC). After centrifuged at 3200 rpm for 10 min, the serum ALT levels were measured.

### AT1-AA affinity purification

On the basis of ELISA detection results, AT1-AA-positive serum (antibody titer exceeding 1:1280) was chosen. IgG (containing AT1-AA) was purified from the sera of pregnant woman with HELLP syndrome by utilizing the MabTrap™ kit (Amersham Biosciences, Piscataway, NJ) according to the manufacturer’s protocol. Antibody titers to AT1-AA were confirmed by an ELISA assay as described above. As control, IgGs from the control group were prepared by using the same procedure.

### Introduction of the antibody into rats

All studies were performed in 230 to 250 g timed-pregnant Sprague Dawley (SD) rats. Animals were housed in a temperature controlled room with a 12:12 h light–dark cycle with free access to food and water.

AT1-AA (100 μL PBS, titer >1:640) was administered intravenously via the tail vein at on day 10 of pregnancy and again at next day 11 (term = 22–23 days) to induce HELLP (Group H, n = 6). After AT1-AA injection, some pregnant rats also received losartan (10 mg/kg/day by gavage) for a period of 10 days (Group T, n = 6). Part of the pregnant rats infused with IgGs from the control group served as controls (Group C, n = 6).

On GD 19, all rats were placed in metabolic cages for 24-h urine collection. On GD 20, the rats had been anesthetized (sodium pentobarbital 50 mg/kg intraperitoneally), and mean arterial pressure (MAP) was measured by common carotid arterial catheterization as described previously^27^. Blood and maternal tissues were immediately collected for future analysis. The biochemical components of HELLP syndrome were measured in plasma and whole blood with respect to hemolysis (LDH and bilirubin), elevated liver enzyme (AST), and platelet levels. Subsequently, all pregnant rats were cesarean section, the numbers of live and dead fetal rats and the body weight of the live rats were record.

### Histological observation

The right part of the liver tissues were fixed in buffered formaldehyde, paraffin embedded, sliced to 4 μm sections, and H&E stained as our previously descrtibed^26^. For ultrastructural analysis, the other part of the liver (1-mm^3^ in size) was fixed with 2.5% and embedded in epoxy resin. Sections stained with uranyl solution were examined under a Philips CM120 transmission electron microscope.

### Detection of mitochondrial membrane potential

After successful anesthesia of the animal with pentobarbital, the abdomen was opened along the abdominal median line to expose the portal vein for catheterization. The liver was perfused quickly with 37 °C D-Hank’s solution at a flow rate of 30 ml/min for 10 min, and then with 0.05% collagenase IV at a flow rate of 5 ml/min for 20 min. The digested liver was then removed, dispersed in pre-cooled D-Hank’s solution, washed with high-glucose DMEM three times, centrifuged at 500 rpm for 5 min to obtain purified hepatocytes. After addition of final-titer 1:160 AT1-AA and 5 mg/L final-concentration Rhodamine 123, the liver cell suspension was incubated at 37 °C for 30 min, washed with PBS twice, and detected by flow cytometry. Data were analyzed using Cell Quest Software, and the results are expressed as percentages of the fluorescence values obtained for control (untreated) hepatocytes.

### Statistical analysis

Results are expressed as means ± SD. Data were analysed by t-test. P-values < 0.05 were considered significant.
